# Overcoming the challenges of iris scanning to identify minors (1–4 years) in the real-world setting

**DOI:** 10.1186/s13104-019-4485-8

**Published:** 2019-07-22

**Authors:** Serge Masyn, Anneleen Vuchelen, Eva Santermans, Freya Rasschaert, Allieu Bangura, Wim Parys, Romain Rutten

**Affiliations:** 1 Johnson & Johnson Global Public Health, Beerse, Belgium; 2 CMAST BVBA, Contractor to Johnson & Johnson Global Public Health, Temse, Belgium; 3 Janssen Research & Development, Beerse, Belgium; 4 Janssen Research & Development, Merksem, Belgium; 5 World Vision, Freetown, Sierra Leone; 6 Janssen Pharmaceutica N.V., Turnhoutseweg 30, 2340 Beerse, Belgium

**Keywords:** Biometrics, Fingerprinting, Imaging, Infants, Iris

## Abstract

**Objective:**

Biometric identification techniques for pediatric use are limited. This investigation studied iris scanning in minors aged 1–4 in two exploratory studies in Belgium (n = 197) and Sierra Leone (n = 230), and in a subsequent clinical study in Sierra Leone (n = 635). Images of participants’ irises were captured using a camera, while a survey assessed the ease of use with children.

**Results:**

The image capture success rate per individual was high; 86.0% of the participants had ≥ 2 successful captures. Iris scan quality and surface were similar in all age groups and in the matching population database. When including feasibility in the analysis of minors aged 3–4, sensitivity and specificity were non-inferior compared to using the biometric of a guardian. However, the quality of iris scanning in minors aged 1–4 was worse than the iris scanning reference quality in adults. A mean total usability score of 1.55 ± 0.27 was calculated; a usability threshold of 1.45 is required for routine use. Overall, this technique is feasible in minors aged 3–4, replacing the use of guardian biometrics. Additional work is ongoing to improve this technique further, striving for uniformity from the age of 1.

**Electronic supplementary material:**

The online version of this article (10.1186/s13104-019-4485-8) contains supplementary material, which is available to authorized users.

## Introduction

In the developed world, national identification numbers are predominantly used to identify patients [1]. However, in many countries these are unavailable, requiring novel techniques for patient identification. New cost-effective and practical options are therefore being developed, such as biometric identification [2, 3]. A successful biometric tool should be easy to operate, capable of identifying entire populations, suitable for the developed and developing world, able to generate reproducible results, and cost-effective.

Current biometric identification methods include fingerprinting, facial recognition, iris scanning, and voice recognition [4–7]. Fingerprint identification is the furthest developed, affordable and easy to use. Hence, it has been successfully implemented for use with adults in healthcare systems globally [8–11].

Patient identification during early childhood is important as most vaccinations schedules initiated at this stage [12, 13]. However, fingerprint identification is suboptimal in young children as they have a shorter distance between structural ridges in the fingerprint compared with later life, make the fingerprint indistinct. As children grow, the ridge distances increase and the scanning device will eventually not be able to identify the same individual [11, 14].

As chaotic morphogenesis occurs in utero, distinctive iris patterns remain the same throughout life [15]. Therefore, this technique may be suitable for identification of children [9, 16]. It is affordable, and, being a contact-free process, may prevent transmission of infectious disease [17–19]. However, iris scanning methodology is currently limited and identification of patients aged 1–4 years relies upon the biometrics of a parent/guardian [9].

We conducted two exploratory studies and one larger clinical study to determine the usability of iris scanning through assessing accuracy of identifying children aged 1‒4 years.

## Main text

### Exploratory studies

Two exploratory, open-label, methodological studies were performed; one in Belgium (June–November 2016) and one in Sierra Leone (March–May 2017). Healthy children were recruited from daycare centers, pre-schools, health centers, and an event for children.

Ethical approval was obtained from the independent ethics committees/institutional review boards in each country. Legal guardians of all children provided written informed consent. Both studies were performed in accordance with current International Council on Harmonization guidelines on Good Clinical Practice, applicable regulatory and country-specific requirements, and the Declaration of Helsinki.

The studies were carried out by Johnson & Johnson’s Clinical Pharmacology Unity and World Vision Sierra Leone. Image capture and data matching were performed as described for the clinical study. Usability and feasibility of the procedure were assessed using weighted five-point scales. Adverse events and safety information reported by investigators were recorded.

### Clinical study

#### Study population

Children were recruited from a community pre-school and three primary healthcare units in Sierra Leone (June–December 2017). Inclusion criteria included minors 1–4 years old (inclusive); able to open eyes; no recent eye infections; and not receiving eye medication.

#### Equipment for procedure

The study was performed by World Vision Sierra Leone. The iris scanning procedure was performed using an IriShield MK 2120 monocular camera (Iritech, Inc.), and a Galaxy Tab S2 8″ 32 GB BT tablet and Neurotechnology iris scanning algorithms.

#### Image capture

An initial capture of each iris was conducted for biometric registration. A second image pair was then captured for recognition at the same visit. The camera was held approximately 5 cm from the child’s eye and automatically captured the image once the iris was in focus.

#### Data matching

Successful iris scans were assessed for quality and surface. Quality was defined as how well a recognition template was created from the raw image, proprietary to the camera manufacture. Surface was a measure of how much surface of the iris could be used for the template. A quality threshold of 70, based on our experience in adults and to preserve a single methodology, was applied to produce good quality images whilst reducing the need for frequent recaptures.

To analyze image data, sensitivity and specificity was measured. Sensitivity measured the proportion of correctly accepted enrolled/registered patients, and specificity the proportion of correctly rejected participants who were not enrolled/registered.

Authenticity between first and second image pairs was ensured by capturing both in one session assigning one subject ID. To assess sensitivity (specificity), the second pair is matched against the full data set of first pairs, including (excluding) the individual under investigation.

#### Usability and feasibility of the procedure

Usability of the device and cooperation of the child were assessed using five survey questions (Additional file 1: Table S1). An additional five-point survey was conducted to obtain feedback on the ease-of-use on a scale of 1 = difficult to 5 = easy, with the opportunity to provide written comments.

Overall usability was calculated using a weighted sum of scores for survey questions and the five-point question. Arbitrarily, a ‘not usable’ result was defined as ≥ 3 ‘Yes’ responses and a score of < 3 on the difficulty scale. The lowest aggregate score was 1.45, which was defined as the usability threshold. Participants in whom matching could not be performed were included as failures.

Usability scores were analyzed across all participants, and for each age category. Feasibility was assessed based on the number of participants who were not included due to an insufficient number of images.

#### Statistics and data analysis

Statistical analyses were performed using a logistic model or generalized estimating equations, including age as a continuous covariate.

Usability survey data were summarized using descriptive statistics, overall and by age category. Comments were collected verbatim.

Sensitivity, specificity, and feasibility were assessed using a logistic model with age as categorical covariate. Sensitivity and specificity were compared to the current standard of identification using non-inferiority testing with a 5% threshold. The quality of iris recognition in adults was also compared in a superiority test.

#### Safety and tolerability

All adverse events and any safety information spontaneously reported by an investigator were recorded.

### Results

#### Exploratory studies

427 children were recruited into two exploratory studies (n = 197 in Belgium; n = 230 in Sierra Leone). The number of iris scans captured was 1660 in Belgium and 2109 in Sierra Leone. Overall, 22.4% and 39.4% of these were successful. In Belgium, 46.2% of participants had ≥ 2 images captured successfully. In Sierra Leone, the success rate was substantially higher, with 90.4% of participants having ≥ 2 successful images captured.

In both studies, the percentage of successful images, proportion of individuals with ≥ 2 successful images and the number of children eligible for matching increased with age.

Compared with standard identification of infants using a parent/guardian biometric, specificity was non-inferior within a 5% threshold for all age groups. Sensitivity was also non-inferior among participants aged 2–4 years in Belgium, but fell outside of the accepted range for non-inferiority in Sierra Leone. As the feasibility of iris scan imaging was very low, non-inferiority compared with the current approach could not be concluded for sensitivity and specificity in Belgium and for sensitivity in Sierra Leone; specificity results were high enough in Sierra Leone in participants aged 2–4 years.

In Belgium, there was frequently a requirement of physical contact, multiple iris captures, and help from a guardian during the scanning process. Some operators also reported that the device made contact with the participant or that capturing an image of the iris was impossible due to the child being afraid of the device. The mean usability score of 1.3308 was below the threshold of 1.45 required for routine use. However, mean scores were above the usability threshold in those aged 3 and 4 years (1.53 and 1.68, respectively). Corresponding data were not available from Sierra Leone due to large variability and poor quality of survey sheets. Learnings from these studies were used in designing the clinical study, including improved operator training and positioning of the iris image, and an increased quality assessment threshold.

#### Clinical study

##### Baseline characteristics

635 healthy children were recruited into the study; 71% were aged 1–2 years. No adverse events were reported, and no participants withdrew due to safety reasons.

##### Iris scan capture—procedure performance

**Fig. 1:**
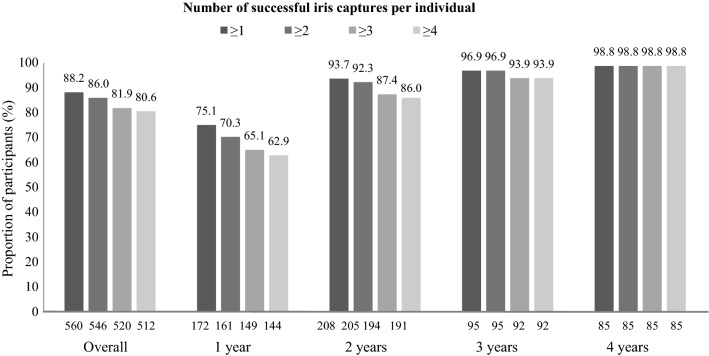
Number of successful iris scans per individual in infants in Sierra Leone (n = 635). n is provided at the bottom of each bar

14,958 iris scans were captured and 2138 (14.3%) were successful (Additional file 2: Figure S1). The number of successful images per individual was high; 86.0% of participants had ≥ 2 successful images captures and 81% successfully had four images captured (Fig. 1).

##### Effect of age on iris scan capture performance

The percentage of successful images captured increased with age, from 10% in 1-year-olds to 24% in aged 4-year-olds (Additional file 2: Figure S1). The proportion of individuals with ≥ 2 successful images also increased with age (*p *< 0.0001; Fig. 1).

##### Quality and surface values of iris scans

The overall quality and surface value of iris scans were similar over the age range studied (Additional file 3: Figure S2a). Similar quality and surface values of iris scans were seen in the slightly smaller subset in the matching database (Additional file 3: Figure S2b).

##### Matching procedure

81% of participants were eligible for the matching procedure. The number of eligible children increased with age (1 year: 62.8%; 2 years: 86.0%; 3 years: 93.9%; 4 years: 99%; *p *< 0.0001).

##### Specificity and sensitivity comparison to other approaches

Across all age groups, sensitivity and specificity were similar, and non-inferior to current methods. Compared with the quality of iris scans in adults, overall specificity and sensitivity were less accurate across all age groups (*p *< 0.0001), except for sensitivity of 3-year-olds in the matching population (*p *= 1.000). The results of the logistic model in which age and study were covariates are shown in Table 1.

**Table 1:** Sensitivity and specificity analysis (clinical study; n = 635 [Sierra Leone])—logistic model

Age	Estimate (95% CI)	*p* value vs infant identification using parent/guardian performance accuracy	*p* value vs adult performance accuracy
Sensitivity in matching population			
1 year	0.896 (0.834–0.936)	0.0028	< 0.0001
2 years	0.932 (0.886–0.960)	< 0.0001	< 0.0001
3 years	1.0000 (0.961–1.000)	< 0.0001	1.0000^a^
4 years	0.988 (0.921–0.998)	0.0013	< 0.0001
Specificity in matching population			
1 year	0.993 (0.952–0.999)	0.0002	< 0.0001
2 years	0.948 (0.905–0.972)	< 0.0001	< 0.0001
3 years	0.946 (0.876–0.977)	0.0007	<0.0001
4 years	0.918 (0.837–0.960)	0.0050	< 0.0001
Sensitivity in enrolled participants (instances in which the matching procedure could not be performed included as failures)			
1 year	0.563 (0.498–0.626)	1.0000	< 0.0001
2 years	0.802 (0.744–0.849)	0.4955	< 0.0001
3 years	0.939 (0.870–0.972)	0.0008	< 0.0001
4 years	0.977 (0.912–0.994)	0.0005	< 0.0001
Specificity in enrolled participants (instances in which the matching procedure could not be performed included as failures)			
1 year	0.624 (0.560–0.685)	1.0000	< 0.0001
2 years	0.815 (0.759–0.861)	0.3030	< 0.0001
3 years	0.888 (0.809–0.937)	0.0178	< 0.0001
4 years	0.907 (0.825–0.953)	0.0088	< 0.0001

Analysis based on logistic models for sensitivity and specificity with age as covariate

^a^Exact testing was performed for sensitivity in 3-year-olds

##### Feasibility analysis

Feasibility was different between the younger (1-to-2-year-old) and older (3-to-4-year-old) infants. As such, non-inferiority compared with the current approach could only be concluded within the older age group, for both sensitivity and specificity (Table 1).

##### Usability

Physical contact with the operator was required for 379 participants (60%), while four (1%) were in contact with the device. Multiple captures were required for 299 participants (48%), and help was needed from the accompanying adult in 52% of cases. 98 participants (16%) were afraid of the device, making iris image capturing impossible. Overall, mean difficulty rating for use of the device was 2.36 ± 1.11.

**Fig. 2:**
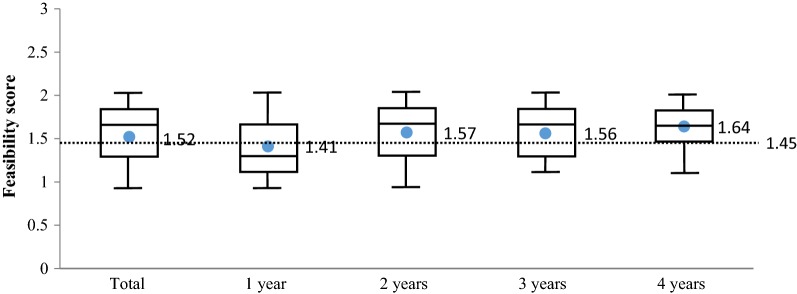
Usability scores according to participant age, assessed using operator survey results (clinical study; n = 635 [Sierra Leone]). Box and whiskers plots indicating median (horizontal line), upper and lower quartiles (boxes), and range (whiskers). Numerical values provided within each box represent the mean. The horizontal dashed line represents the usability threshold (1.45). The number of survey responses is provided at the base of each plot

The mean (total) usability score of 1.55 ± 0.27 was above the 1.45 usability threshold required for routine use. The highest score was observed in 4-year-olds (1.64 ± 0.22; 4); and was below the usability threshold for 1-year-olds (1.41 ± 0.28) (Fig. 2).

### Discussion

We aimed to address the critical need for accurate identification of minors to ensure they receive correct vaccination schedules, based on the potential benefits of iris scanning [9, 16, 20]. This clinical study shows that iris scanning was non-inferior to current standard identification approaches in children aged 3–4.

The exploratory studies demonstrated the potential of iris scanning in children, but highlighted technical issues that were subsequently resolved for the clinical study. Changes included improved operator training, controlled environmental conditions, and a higher quality threshold.

Nevertheless, issues were encountered in the clinical study, which must yet be overcome for iris scanning technology to become a clinically applicable method of identification with children. The iris is small and can be obscured, which can impede capture. Difficulties also occur when obtaining a scan in non-cooperative infants—the iris is a ‘moving’ target that must remain motionless for capture, and operators needed multiple captures per eye in many instances. This may be overcome by new technologies that can capture the iris in motion.

One of the major drivers for the investigation of iris scanning was that it would avoid contact, thus mitigating transmission of diseases [9]. However, technology operators completing the survey also noted that they made physical contact with the participant ≥ 50% of the time.

### Conclusions

We have documented evidence on the age threshold at which iris scanning can be used in minors instead of using the biometrics of a parent/guardian. We can conclude that iris scanning is non-inferior in children aged 3–4 years, allowing for uniformity of the process. For younger children, several outstanding issues need to be addressed. However, this procedure has clear potential to identify minors, with wide-scale application in the developing world.

## Limitations

- Four images were necessary to perform matching; in a real-world setting, two images per visit would be sufficient.
- Feasibility may have been underestimated.

## Additional files

**Additional file 1: Table S1.** Usability survey questions answered by biometric operators for each participant.

**Additional file 2: Figure S1.** Iris scanning capture in infants in Sierra Leone (n = 569). 66 participants without age data are omitted from overall data in Fig. 1. n is provided at the bottom of each bar.

**Additional file 3: Figure S2.** (a) Quality and surface of successful iris captures in enrolled participants in Sierra Leone (2138 images), (b) quality and surface of recognition iris scans in the matching population database (2048 images) across the different age range (1‒4 years) in Sierra Leone. Box and whiskers plots indicating median (horizontal line), upper and lower quartiles (boxes), and range (whiskers), with outliers plotted as individual points. Numerical values provided within each box represent the mean. The number of images is provided at the base of each bar.

## Data Availability

The data sharing policy of Janssen Pharmaceutical Companies of Johnson & Johnson is available at https://www.janssen.com/clinical-trials/transparency. As noted on this site, requests for access to the study data can be submitted through Yale Open Data Access (YODA) Project site at http://yoda.yale.edu.

## References

[1] Beynon-Davies P (2006). Personal identity management in the information polity: the case of the UK national identity card. Inf Polity.

[2] Storisteanu DM, Norman TL, Grigore A, Norman TL (2015). Biometric fingerprint system to enable rapid and accurate identification of beneficiaries. Glob Health Sci Pract.

[3] Unar J, Seng WC, Abbasi A (2014). A review of biometric technology along with trends and prospects. Pattern Recognit.

[4] Jain AK, Ross A, Prabhakar S (2004). An introduction to biometric recognition. IEEE Trans Circuits Syst Video Technol.

[5] Grother P, Matey JR, Tabassi E, Quinn GW, Chumakov M. Temporal stability of iris recognition accuracy, IREX VI K National Institute of Standards and Technology (NIST) Interagency Report 7948. 2013. http://nvlpubs.nist.gov/nistpubs/ir/2013/NIST.IR.7948.pdf. Accessed 4 Apr 2019.

[6] Jain AK, Arora SS, Best-Rowden L, Cao K. Biometrics for child vaccination and welfare: persistence of fingerprint recognition for infants and toddlers. 2015. http://biometrics.cse.msu.edu/Publications/Fingerprint/Jainetal_BiometricsChildVaccinationWelfare_MSUTechRepMSU-CSE-15-7.pdf. Accessed 4 Apr 2019.

[7] Yoon S, Jain AK (2015). Longitudinal study of fingerprint recognition. Proc Natl Acad Sci USA.

[8] Ross A, Jain A, Reisman J (2003). A hybrid fingerprint matcher. Pattern Recognit.

[9] Saini R, Rana N (2014). Comparison of various biometric methods. Int J Adv Sci Eng Technol.

[10] Cresswell KM, Sheik A (2008). Information technology–based approaches to reducing repeat drug exposure in patients with known drug allergies. J Allergy Clin Immunol.

[11] Odei-Lartey E, Boateng D, Danso S, Kwarteng A, Abokyi L, Amenga-Etego S (2016). The application of a biometric identification technique for linking community and hospital data in rural Ghana. Glob Health Action.

[12] United Nations. Millennium development goals and beyond. 2015. http://www.un.org/millenniumgoals/pdf/Goal_4_fs.pdf. Accessed 4 Apr 2019.

[13] Jain AK, Cao K, Arora SS. Recognizing infants and toddlers using fingerprints: increasing the vaccination coverage. In: Proceedings of the international joint conference on biometrics, clearwater, FL, USA. 2014. https://ieeexplore.ieee.org/document/6996252. Accessed 17 Apr 2019.

[14] European Commission Joint Research Centre. Fingerprint recognition for children. Final report. Institute for the Protection and Security of the Citizen and Digital Citizen Security Unit. 2013. http://publications.jrc.ec.europa.eu/repository/bitstream/JRC85145/fingerprint%20re%20for%20children%20final%20report%20%28pdf%29.pdf. Accessed 4 Apr 2019.

[15] Daugman JG (1993). High confidence visual recognition of persons by a test of statistical independence. IEEE Trans Pattern Anal Mach Intell.

[16] Davis-Silberman N, Ashery-Padan R (2007). Iris development in vertebrates; genetic and molecular considerations. Brain Res.

[17] Giusti M. ‘Golden Age’ for iris recognition? SecureID news, AVISIAN Publications. https://www.secureidnews.com/news-item/golden-age-for-iris-recognition/. Accessed 4 Apr 2019.

[18] World Health Organization. Ebola virus disease fact sheet. 2018. http://www.who.int/mediacentre/factsheets/fs103/en/. Accessed 4 Apr 2019.

[19] Jacobs JA, Van Ranst M (2008). Biometric fingerprinting for visa application: device and procedure are risk factors for infection transmission. J Travel Med.

[20] Daugman JG (2007). New methods in iris recognition. IEEE Trans Syst Man Cybern B Cybern.

