# Supplementary material for: Overcoming the challenges of iris scanning to identify minors (1–4 years) in the real-world setting
Source: BMC Res Notes. 2019 Jul 22;12:448. doi: 10.1186/s13104-019-4485-8 (PMC6647056; doi:10.1186/s13104-019-4485-8)
Supplement: Supplementary file 1 — Additional file 1: Table S1. Usability survey questions answered by biometric operators for each participant. [file 13104_2019_4485_MOESM1_ESM.docx]

**Additional Materials**

**Table S1**

| **Question** | **Possible answers** |
| --- | --- |
| Did you, the operator, need to make physical contact with the participant? | Yes/No |
| Did the device make contact with the participant? | Yes/No |
| Did you, the operator, require multiple captures for a given eye? | Yes/No |
| Was the participant afraid of the device, making capturing the iris impossible? | Yes/No |
| Was any help required from the accompanying adult? | Yes/No |
