# Supplementary material for: Overcoming the challenges of iris scanning to identify minors (1–4 years) in the real-world setting
Source: BMC Res Notes. 2019 Jul 22;12:448. doi: 10.1186/s13104-019-4485-8 (PMC6647056; doi:10.1186/s13104-019-4485-8)
Supplement: Supplementary file 2 — Additional file 2: Figure S1. Iris scanning capture in infants in Sierra Leone (n = 569). 66 participants without age data are omitted from overall data in Fig. 1. n is provided at the bottom of each bar. [file 13104_2019_4485_MOESM2_ESM.pptx]

## Slide 1
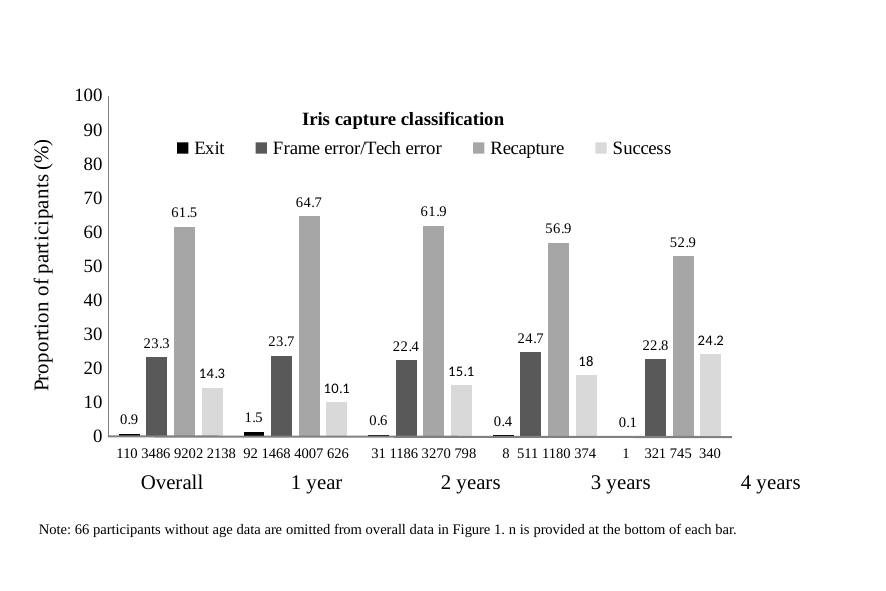

### Chart
| Category | Exit | Frame error/Tech error | Recapture | Success |
|---|---|---|---|---|
| Total | 0.9 | 23.3 | 61.5 | 14.3 |
| 1 year | 1.5 | 23.7 | 64.7 | 10.1 |
| 2 years | 0.6 | 22.4 | 61.9 | 15.1 |
| 3 years | 0.4 | 24.7 | 56.9 | 18.0 |
| 4 years | 0.1 | 22.8 | 52.9 | 24.2 |Iris capture classification
110 3486 9202 2138 92 1468 4007 626 31 1186 3270 798 8 511 1180 374 1 321 745 340
Overall	1 year	2 years	3 years	4 years
Note: 66 participants without age data are omitted from overall data in Figure 1. n is provided at the bottom of each bar.
