# Supplementary material for: Overcoming the challenges of iris scanning to identify minors (1–4 years) in the real-world setting
Source: BMC Res Notes. 2019 Jul 22;12:448. doi: 10.1186/s13104-019-4485-8 (PMC6647056; doi:10.1186/s13104-019-4485-8)
Supplement: Supplementary file 3 — Additional file 3: Figure S2. (a) Quality and surface of successful iris captures in enrolled participants in Sierra Leone (2138 images), (b) quality and surface of recognition iris scans in the matching population database (2048 images) across the different age range (1‒4 years) in Sierra Leone. Box and whiskers plots indicating median (horizontal line), upper and lower quartiles (boxes), and range (whiskers), with outliers plotted as individual points. Numerical values provided within each box represent the mean. The number of images is provided at the base of each bar. [file 13104_2019_4485_MOESM3_ESM.pptx]

## Slide 1
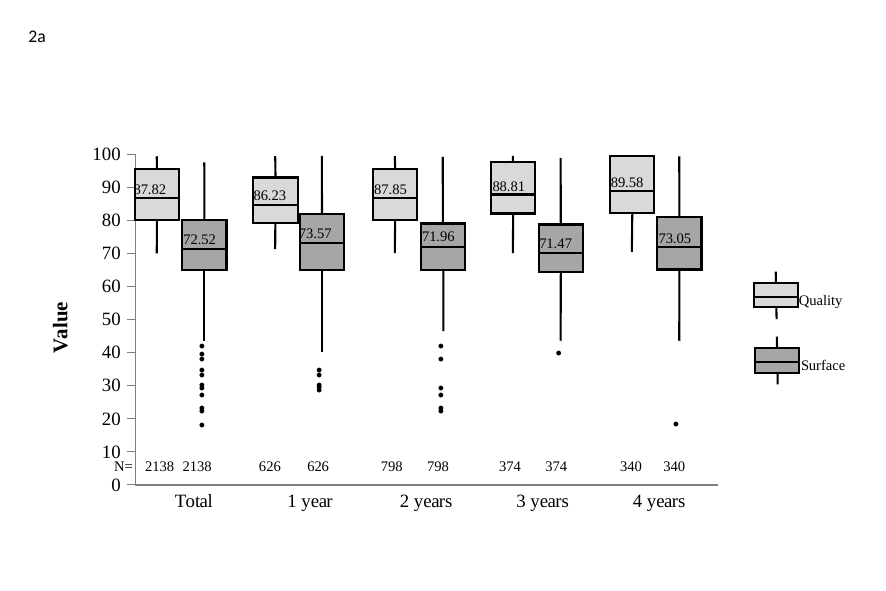

2a
### Chart
| Category | Quality | Surface |
|---|---|---|
| Total | 87.82 | 72.52 |
| 1 year | 86.23 | 73.57 |
| 2 years | 87.85 | 71.9 |
| 3 years | 88.81 | 71.47 |
| 4 years | 89.52 | 73.05 |
73.05
●
89.58
88.81
87.82
87.85
86.23
73.57
71.96
72.52
71.47
Quality
●
●
●
●
●
●
●
●
●
●
●
●
●
●
●
●
●
Surface
●
●
●
●
●
●
626
798
N=
2138
2138
626
798
374
374
340
340

## Slide 2
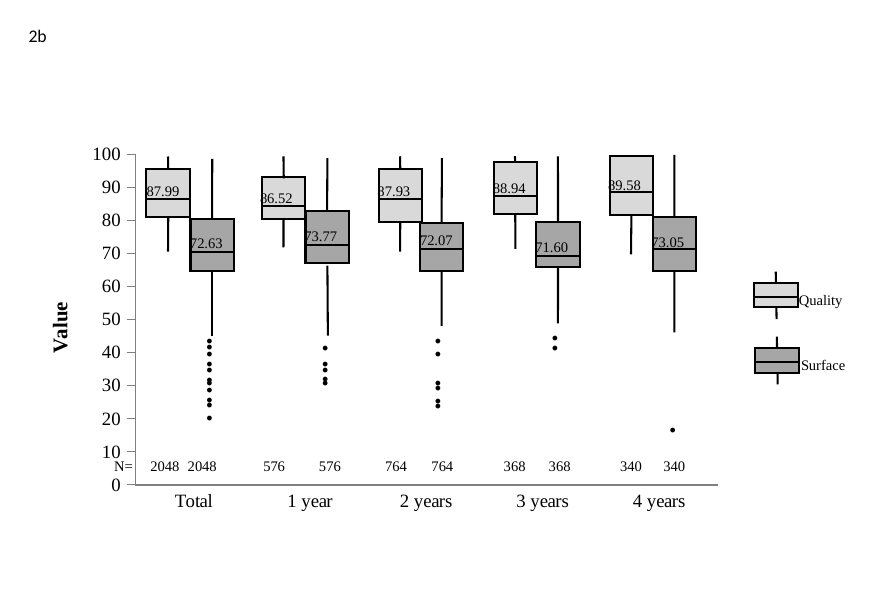

2b
### Chart
| Category | Quality | Surface |
|---|---|---|
| Total | 87.82 | 72.52 |
| 1 year | 86.23 | 73.57 |
| 2 years | 87.85 | 71.9 |
| 3 years | 88.81 | 71.47 |
| 4 years | 89.52 | 73.05 |
89.58
88.94
87.99
87.93
86.52
73.77
72.07
73.05
72.63
71.60
Quality
●
●
●
●
●
●
●
●
●
●
●
●
Surface
●
●
●
●
●
●
●
●
●
●
●
●
●
576
764
N=
2048
2048
576
764
368
368
340
340
